# Supplementary material for: Trends in suicide among adolescents aged 14–17 years in India: 2014–2019
Source: Glob Ment Health (Camb). 2025 Jul 31;12:e90. doi: 10.1017/gmh.2025.10044 (PMC12394019; doi:10.1017/gmh.2025.10044)
Supplement: Arya et al. supplementary material [file S2054425125100447sup001.docx]

Table of Contents

[Table 1: Adolescent suicide rates (crude) (with 95% confidence interval (CI)) aged 14-17 years in India by state (both sexes combined) 3](#_Toc179297428)

[Table 2: Adolescent suicide rates (crude) (with 95% confidence interval (CI)) aged 14-17 years in India by state (Male) 4](#_Toc179297429)

[Table 3: Adolescent suicide rates (crude) (with 95% confidence interval (CI)) aged 14-17 years in India by state (Female) 5](#_Toc179297430)

[Table 4: Suicide rate ratios for 2017-2019 versus 2014-2016 (with 95% uncertainty interval (UI)) by sex and state 6](#_Toc179297431)

## Table 1: Adolescent suicide rates (crude) (with 95% confidence interval (CI)) aged 14-17 years in India by state (both sexes combined)

| **State** | **2014** | **2015** | **2016** | **2017** | **2018** | **2019** |
| --- | --- | --- | --- | --- | --- | --- |
| Andhra Pradesh | 18.28 (16.95-19.70) | 15.34 (14.11-16.64) | 16.87 (15.57-18.25) | 17.82 (16.48-19.25) | 16.83 (15.52-18.23) | 18.25 (16.87-19.72) |
| Arunachal Pradesh | 13.51 (8.13-21.10) | 18.18 (11.87-26.64) | 9.65 (5.27-16.20) | 3.40 (1.10-7.95) | 9.46 (5.17-15.87) | 4.69 (1.88-9.67) |
| Assam | 14.62 (13.21-16.13) | 12.84 (11.54-14.26) | 9.99 (8.84-11.24) | 7.25 (6.29-8.33) | 7.06 (6.11-8.12) | 7.27 (6.31-8.33) |
| Bihar | 0.66 (0.51-0.84) | 0.83 (0.67-1.03) | 0.69 (0.54-0.86) | 0.87 (0.71-1.07) | 0.94 (0.76-1.14) | 1.30 (1.10-1.53) |
| Chhattisgarh | 18.25 (16.56-20.06) | 21.50 (19.68-23.45) | 19.76 (18.02-21.62) | 19.37 (17.66-21.20) | 22.53 (20.68-24.49) | 27.19 (25.17-29.33) |
| Delhi | 15.77 (13.75-18.00) | 10.99 (9.31-12.88) | 14.82 (12.86-17.00) | 17.67 (15.51-20.04) | 16.86 (14.75-19.19) | 15.08 (13.09-17.29) |
| Goa | 8.73 (3.77-17.21) | 12.14 (6.06-21.72) | 15.61 (8.53-26.20) | 11.26 (5.40-20.70) | 17.04 (9.53-28.10) | 17.18 (9.61-28.33) |
| Gujarat | 9.01 (8.19-9.88) | 9.52 (8.69-10.42) | 9.97 (9.11-10.89) | 9.07 (8.25-9.95) | 9.63 (8.79-10.53) | 8.76 (7.96-9.62) |
| Haryana | 8.21 (7.06-9.49) | 7.69 (6.58-8.93) | 10.78 (9.46-12.24) | 9.61 (8.36-11.00) | 9.44 (8.19-10.81) | 9.03 (7.81-10.38) |
| Himachal Pradesh | 5.19 (3.42-7.56) | 5.04 (3.29-7.38) | 7.22 (5.08-9.95) | 7.85 (5.61-10.69) | 12.24 (9.38-15.69) | 8.33 (6.00-11.26) |
| Jammu & Kashmir | 1.93 (1.21-2.92) | 1.81 (1.12-2.77) | 1.10 (0.58-1.89) | 1.25 (0.70-2.07) | 1.15 (0.63-1.94) | 0.82 (0.39-1.51) |
| Jharkhand | 5.91 (5.08-6.84) | 2.25 (1.76-2.85) | 3.27 (2.67-3.96) | 5.73 (4.93-6.63) | 8.03 (7.08-9.07) | 9.77 (8.72-10.91) |
| Karnataka | 16.37 (15.24-17.57) | 13.13 (12.11-14.21) | 8.31 (7.50-9.18) | 10.70 (9.78-11.69) | 10.41 (9.50-11.39) | 10.10 (9.20-11.06) |
| Kerala | 12.96 (11.48-14.58) | 12.23 (10.78-13.81) | 9.67 (8.38-11.09) | 11.19 (9.80-12.73) | 10.56 (9.21-12.06) | 11.82 (10.38-13.41) |
| Madhya Pradesh | 13.23 (12.38-14.12) | 12.88 (12.05-13.75) | 13.31 (12.47-14.20) | 14.81 (13.92-15.74) | 13.96 (13.11-14.86) | 15.16 (14.27-16.10) |
| Maharashtra | 8.83 (8.22-9.48) | 8.38 (7.78-9.01) | 10.26 (9.60-10.96) | 9.95 (9.30-10.64) | 9.53 (8.88-10.20) | 10.01 (9.35-10.71) |
| Manipur | 3.52 (1.61-6.68) | 1.53 (0.41-3.94) | 1.51 (0.41-3.88) | 1.12 (0.23-3.27) | 3.68 (1.76-6.77) | 2.18 (0.80-4.74) |
| Meghalaya | 3.75 (1.87-6.72) | 5.70 (3.32-9.12) | 4.94 (2.76-8.14) | 5.17 (2.95-8.40) | 6.04 (3.63-9.43) | 4.69 (2.62-7.73) |
| Mizoram | 4.26 (1.16-10.92) | 15.86 (8.88-26.17) | 5.23 (1.69-12.21) | 12.41 (6.41-21.69) | 2.04 (0.24-7.38) | 5.04 (1.63-11.76) |
| Nagaland | 1.11 (0.13-4.03) | 1.69 (0.34-4.95) | 1.13 (0.13-4.11) | 0.57 (0.01-3.19) | 1.72 (0.35-5.05) | 2.89 (0.94-6.76) |
| Odisha | 14.51 (13.25-15.86) | 9.36 (8.35-10.45) | 10.09 (9.04-11.22) | 8.59 (7.62-9.64) | 9.06 (8.07-10.14) | 12.26 (11.10-13.51) |
| Punjab | 2.78 (2.12-3.57) | 2.03 (1.47-2.72) | 2.33 (1.73-3.07) | 2.40 (1.79-3.16) | 3.14 (2.43-4.00) | 3.31 (2.58-4.19) |
| Rajasthan | 4.64 (4.13-5.19) | 3.77 (3.32-4.26) | 3.94 (3.48-4.45) | 4.19 (3.71-4.70) | 4.70 (4.20-5.25) | 4.97 (4.45-5.53) |
| Sikkim | 43.79 (27.76-65.70) | 34.77 (20.60-54.95) | 35.31 (20.92-55.8) | 25.92 (13.80-44.33) | 28.36 (15.50-47.59) | 28.77 (15.73-48.27) |
| Tamil Nadu | 18.38 (17.22-19.61) | 16.89 (15.77-18.07) | 14.96 (13.90-16.08) | 14.43 (13.38-15.53) | 13.66 (12.65-14.74) | 13.41 (12.40-14.48) |
| Telangana | 12.17 (10.93-13.51) | 10.34 (9.19-11.59) | 11.06 (9.87-12.36) | 13.10 (11.79-14.52) | 14.30 (12.92-15.79) | 12.82 (11.50-14.25) |
| Tripura | 33.02 (26.75-40.32) | 18.29 (13.70-23.92) | 19.03 (14.33-24.77) | 29.46 (23.53-36.43) | 16.30 (11.98-21.68) | 22.89 (17.71-29.13) |
| Union Territories* (except Delhi) | 20.32 (15.18-26.65) | 21.09 (15.84-27.52) | 17.18 (12.48-23.06) | 22.24 (16.85-28.82) | 28.47 (22.32-35.80) | 18.33 (13.47-24.37) |
| Uttar Pradesh | 1.49 (1.33-1.67) | 1.43 (1.27-1.59) | 1.09 (0.95-1.23) | 1.36 (1.21-1.53) | 1.79 (1.61-1.97) | 1.62 (1.45-1.79) |
| Uttarakhand | 4.00 (2.83-5.49) | 1.46 (0.80-2.46) | 0.52 (0.16-1.21) | 0.93 (0.42-1.78) | 3.44 (2.36-4.83) | 3.54 (2.45-4.95) |
| West Bengal | 20.44 (19.41-21.51) | 14.30 (13.44-15.20) | 11.80 (11.02-12.62) | 14.10 (13.23-15.00) | 13.86 (13.00-14.76) | 11.91 (11.11-12.74) |
| ***Less Developed States*** | ***5.65 (5.46-5.85)*** | ***4.96 (4.78-5.14)*** | ***4.70 (4.53-4.88)*** | ***4.97 (4.79-5.15)*** | ***5.34 (5.16-5.53)*** | ***5.99 (5.80-6.19)*** |
| ***More Developed States*** | ***12.51 (12.19-12.83)*** | ***10.57 (10.28-10.87)*** | ***10.19 (9.90-10.48)*** | ***10.74 (10.45-11.05)*** | ***10.53 (10.23-10.83)*** | ***10.21 (9.92-10.51)*** |
| **India** | **8.74 (8.56-8.92)** | **7.47 (7.31-7.64)** | **7.12 (6.97-7.29)** | **7.51 (7.34-7.67)** | **7.62 (7.46-7.79)** | **7.82 (7.65-7.99)** |

*Union Territories (except Delhi) are not included in less or more developed states categories

## Table 2: Adolescent suicide rates (crude) (with 95% confidence interval (CI)) aged 14-17 years in India by state (Male)

| **State** | **2014** | **2015** | **2016** | **2017** | **2018** | **2019** |
| --- | --- | --- | --- | --- | --- | --- |
| Andhra Pradesh | 17.46 (15.65-19.42) | 12.50 (10.97-14.18) | 17.20 (15.38-19.17) | 15.38 (13.65-17.26) | 13.99 (12.33-15.81) | 15.94 (14.15-17.89) |
| Arunachal Pradesh | 7.10 (2.30-16.59) | 14.00 (6.71-25.75) | 2.76 (0.33-9.98) | 2.73 (0.33-9.88) | 12.20 (5.58-23.17) | 4.03 (0.83-11.80) |
| Assam | 17.20 (15.09-19.53) | 13.83 (11.95-15.93) | 11.03 (9.36-12.9) | 7.42 (6.07-8.98) | 8.49 (7.05-10.15) | 9.00 (7.52-10.70) |
| Bihar | 0.73 (0.52-1.00) | 0.87 (0.64-1.16) | 0.55 (0.37-0.78) | 0.90 (0.67-1.18) | 0.99 (0.75-1.28) | 1.24 (0.97-1.56) |
| Chhattisgarh | 18.08 (15.74-20.68) | 19.89 (17.44-22.58) | 19.87 (17.43-22.55) | 19.73 (17.31-22.39) | 21.73 (19.19-24.50) | 27.83 (24.96-30.94) |
| Delhi | 17.18 (14.35-20.40) | 10.50 (8.31-13.09) | 11.10 (8.84-13.76) | 18.43 (15.47-21.79) | 13.93 (11.37-16.90) | 12.22 (9.83-15.02) |
| Goa | 4.16 (0.50-15.04) | 12.64 (4.64-27.52) | 14.92 (6.00-30.75) | 8.62 (2.34-22.07) | 17.41 (7.51-34.31) | 10.98 (3.56-25.63) |
| Gujarat | 7.45 (6.45-8.57) | 6.72 (5.77-7.78) | 7.80 (6.77-8.94) | 6.81 (5.85-7.88) | 6.99 (6.02-8.07) | 5.28 (4.44-6.23) |
| Haryana | 9.87 (8.19-11.80) | 8.51 (6.95-10.31) | 13.15 (11.19-15.35) | 11.98 (10.11-14.10) | 11.14 (9.33-13.19) | 8.78 (7.18-10.63) |
| Himachal Pradesh | 3.67 (1.76-6.75) | 4.80 (2.55-8.21) | 5.57 (3.12-9.19) | 6.73 (3.98-10.63) | 10.15 (6.68-14.76) | 7.17 (4.32-11.21) |
| Jammu & Kashmir | 1.00 (0.36-2.19) | 0.65 (0.17-1.68) | 1.45 (0.66-2.76) | 1.27 (0.54-2.50) | 0.94 (0.34-2.05) | 1.08 (0.43-2.24) |
| Jharkhand | 6.37 (5.19-7.75) | 2.63 (1.90-3.56) | 3.90 (2.99-4.99) | 7.28 (6.03-8.71) | 10.05 (8.58-11.70) | 12.30 (10.68-14.11) |
| Karnataka | 19.09 (17.39-20.91) | 11.76 (10.43-13.21) | 6.78 (5.78-7.91) | 9.07 (7.89-10.36) | 9.67 (8.46-11.01) | 9.02 (7.85-10.33) |
| Kerala | 10.66 (8.80-12.79) | 12.53 (10.5-14.84) | 7.36 (5.82-9.19) | 9.81 (8.00-11.89) | 10.75 (8.85-12.94) | 10.45 (8.58-12.62) |
| Madhya Pradesh | 11.55 (10.46-12.72) | 10.96 (9.91-12.09) | 9.53 (8.55-10.58) | 11.29 (10.23-12.43) | 10.10 (9.10-11.18) | 11.74 (10.67-12.9) |
| Maharashtra | 6.56 (5.84-7.34) | 6.55 (5.83-7.34) | 8.08 (7.28-8.95) | 8.04 (7.24-8.91) | 7.73 (6.95-8.59) | 8.90 (8.05-9.81) |
| Manipur | 1.54 (0.18-5.57) | 0.75 (0.01-4.22) | 0.74 (0.01-4.15) | 1.46 (0.17-5.30) | 2.89 (0.78-7.40) | 0.00 (0.00-2.62) |
| Meghalaya | 3.40 (1.10-7.94) | 8.03 (4.14-14.02) | 3.28 (1.06-7.67) | 6.46 (3.09-11.88) | 6.34 (3.04-11.67) | 6.86 (3.42-12.28) |
| Mizoram | 2.11 (0.05-11.76) | 16.74 (7.22-32.99) | 4.14 (0.50-14.97) | 18.43 (8.43-35.00) | 2.02 (0.05-11.27) | 1.99 (0.05-11.12) |
| Nagaland | 0.00 (0.00-4.01) | 2.20 (0.26-7.95) | 1.11 (0.02-6.19) | 1.11 (0.02-6.23) | 2.24 (0.27-8.12) | 2.26 (0.27-8.16) |
| Odisha | 16.35 (14.47-18.4) | 8.38 (7.05-9.89) | 9.76 (8.33-11.38) | 6.68 (5.50-8.04) | 7.05 (5.84-8.45) | 11.79 (10.2-13.56) |
| Punjab | 4.03 (2.98-5.33) | 2.58 (1.75-3.66) | 2.02 (1.29-3.01) | 1.96 (1.24-2.94) | 3.10 (2.17-4.29) | 2.95 (2.04-4.13) |
| Rajasthan | 5.06 (4.34-5.86) | 4.06 (3.42-4.78) | 4.41 (3.74-5.15) | 4.79 (4.10-5.57) | 5.05 (4.33-5.84) | 5.19 (4.47-5.99) |
| Sikkim | 33.77 (15.44-64.11) | 38.11 (18.27-70.10) | 23.24 (8.53-50.59) | 31.52 (13.60-62.10) | 24.02 (8.81-52.29) | 24.37 (8.94-53.05) |
| Tamil Nadu | 19.50 (17.83-21.29) | 14.49 (13.05-16.05) | 12.22 (10.90-13.66) | 12.22 (10.90-13.67) | 11.51 (10.22-12.92) | 9.86 (8.66-11.17) |
| Telangana | 12.27 (10.54-14.20) | 7.90 (6.52-9.49) | 12.03 (10.30-13.96) | 9.94 (8.37-11.73) | 12.18 (10.42-14.16) | 10.48 (8.83-12.33) |
| Tripura | 32.71 (24.12-43.37) | 13.67 (8.35-21.11) | 19.18 (12.74-27.72) | 19.90 (13.32-28.58) | 15.79 (10.01-23.70) | 21.97 (15.03-31.02) |
| Union Territories* (except Delhi) | 12.20 (7.10-19.54) | 17.27 (11.06-25.69) | 9.37 (4.99-16.03) | 21.69 (14.63-30.96) | 29.70 (21.31-40.29) | 13.79 (8.30-21.53) |
| Uttar Pradesh | 1.47 (1.25-1.71) | 1.21 (1.02-1.43) | 1.06 (0.88-1.27) | 1.21 (1.02-1.43) | 1.62 (1.40-1.87) | 1.32 (1.12-1.55) |
| Uttarakhand | 6.46 (4.42-9.12) | 1.40 (0.56-2.89) | 0.20 (0.00-1.11) | 0.59 (0.12-1.74) | 2.39 (1.23-4.17) | 1.98 (0.95-3.65) |
| West Bengal | 19.10 (17.71-20.56) | 13.51 (12.34-14.75) | 10.72 (9.68-11.84) | 13.05 (11.89-14.28) | 12.35 (11.22-13.56) | 9.03 (8.06-10.08) |
| ***Less Developed States*** | ***5.64 (5.38-5.91)*** | ***4.55 (4.31-4.79)*** | ***4.23 (4.00-4.46)*** | ***4.48 (4.25-4.72)*** | ***4.83 (4.59-5.07)*** | ***5.59 (5.33-5.85)*** |
| ***More Developed States*** | ***11.97 (11.54-12.41)*** | ***9.16 (8.79-9.55)*** | ***8.82 (8.45-9.20)*** | ***9.39 (9.01-9.79)*** | ***9.09 (8.71-9.48)*** | ***8.30 (7.93-8.67)*** |
| **India** | **8.47 (8.23-8.72)** | **6.61 (6.40-6.82)** | **6.24 (6.03-6.45)** | **6.64 (6.43-6.86)** | **6.71 (6.50-6.93)** | **6.76 (6.54-6.98)** |

*Union Territories (except Delhi) are not included in less or more developed states categories

## Table 3: Adolescent suicide rates (crude) (with 95% confidence interval (CI)) aged 14-17 years in India by state (Female)

| **State** | **2014** | **2015** | **2016** | **2017** | **2018** | **2019** |
| --- | --- | --- | --- | --- | --- | --- |
| Andhra Pradesh | 19.15 (17.20-21.25) | 18.32 (16.41-20.39) | 16.52 (14.69-18.50) | 20.39 (18.34-22.60) | 19.82 (17.79-22.03) | 20.69 (18.59-22.96) |
| Arunachal Pradesh | 19.92 (10.89-33.42) | 22.35 (12.77-36.30) | 16.51 (8.53-28.84) | 4.07 (0.84-11.92) | 6.73 (2.18-15.71) | 5.34 (1.45-13.69) |
| Assam | 11.93 (10.15-13.94) | 11.82 (10.05-13.80) | 8.91 (7.39-10.65) | 7.09 (5.74-8.64) | 5.58 (4.40-6.98) | 5.48 (4.31-6.85) |
| Bihar | 0.58 (0.38-0.84) | 0.79 (0.56-1.08) | 0.84 (0.61-1.14) | 0.84 (0.61-1.13) | 0.88 (0.64-1.17) | 1.37 (1.07-1.72) |
| Chhattisgarh | 18.41 (16.04-21.04) | 23.13 (20.48-26.03) | 19.65 (17.22-22.32) | 19.01 (16.63-21.63) | 23.33 (20.69-26.21) | 26.54 (23.74-29.59) |
| Delhi | 14.08 (11.31-17.33) | 11.58 (9.07-14.56) | 19.25 (15.97-23.00) | 16.76 (13.71-20.29) | 20.33 (16.95-24.19) | 18.46 (15.24-22.15) |
| Goa | 13.78 (5.05-30.00) | 11.59 (3.76-27.05) | 16.37 (6.58-33.73) | 14.14 (5.19-30.79) | 16.63 (6.68-34.27) | 23.93 (11.47-44.00) |
| Gujarat | 10.78 (9.48-12.21) | 12.73 (11.32-14.27) | 12.44 (11.05-13.97) | 11.65 (10.30-13.12) | 12.65 (11.24-14.18) | 12.73 (11.33-14.26) |
| Haryana | 6.18 (4.73-7.92) | 6.68 (5.18-8.49) | 7.91 (6.26-9.85) | 6.73 (5.22-8.55) | 7.37 (5.78-9.27) | 9.34 (7.52-11.45) |
| Himachal Pradesh | 6.88 (4.01-11.02) | 5.30 (2.82-9.07) | 9.04 (5.66-13.69) | 9.10 (5.70-13.78) | 14.55 (10.14-20.24) | 9.61 (6.09-14.41) |
| Jammu & Kashmir | 2.94 (1.68-4.78) | 3.08 (1.79-4.94) | 0.71 (0.19-1.83) | 1.23 (0.49-2.55) | 1.40 (0.60-2.76) | 0.52 (0.10-1.52) |
| Jharkhand | 5.42 (4.30-6.75) | 1.86 (1.23-2.68) | 2.61 (1.86-3.55) | 4.11 (3.16-5.25) | 5.91 (4.77-7.24) | 7.11 (5.86-8.55) |
| Karnataka | 13.49 (12.02-15.08) | 14.58 (13.05-16.23) | 9.92 (8.66-11.31) | 12.43 (11.02-13.98) | 11.20 (9.85-12.68) | 11.23 (9.88-12.71) |
| Kerala | 15.31 (13.05-17.86) | 11.92 (9.92-14.20) | 12.02 (10.00-14.32) | 12.61 (10.53-14.97) | 10.37 (8.49-12.54) | 13.21 (11.07-15.65) |
| Madhya Pradesh | 15.07 (13.77-16.46) | 14.98 (13.69-16.36) | 17.44 (16.06-18.92) | 18.64 (17.21-20.15) | 18.17 (16.76-19.66) | 18.88 (17.45-20.40) |
| Maharashtra | 11.38 (10.38-12.46) | 10.43 (9.46-11.46) | 12.71 (11.64-13.85) | 12.09 (11.04-13.21) | 11.53 (10.51-12.63) | 11.26 (10.24-12.34) |
| Manipur | 5.55 (2.23-11.44) | 2.34 (0.48-6.84) | 2.31 (0.47-6.75) | 0.76 (0.01-4.23) | 4.50 (1.65-9.80) | 4.44 (1.63-9.67) |
| Meghalaya | 4.10 (1.50-8.94) | 3.36 (1.09-7.84) | 6.59 (3.16-12.13) | 3.88 (1.42-8.46) | 5.73 (2.62-10.88) | 2.50 (0.68-6.41) |
| Mizoram | 6.47 (1.33-18.92) | 14.97 (6.01-30.84) | 6.34 (1.30-18.55) | 6.27 (1.29-18.33) | 2.06 (0.05-11.50) | 8.14 (2.21-20.85) |
| Nagaland | 2.29 (0.27-8.28) | 1.15 (0.02-6.46) | 1.16 (0.02-6.51) | 0.00 (0.00-4.34) | 1.18 (0.02-6.59) | 3.56 (0.73-10.42) |
| Odisha | 12.66 (11.02-14.49) | 10.34 (8.85-12.00) | 10.41 (8.92-12.08) | 10.50 (9.00-12.18) | 11.08 (9.54-12.80) | 12.74 (11.08-14.58) |
| Punjab | 1.22 (0.63-2.14) | 1.34 (0.71-2.30) | 2.72 (1.77-3.98) | 2.96 (1.96-4.27) | 3.19 (2.15-4.56) | 3.75 (2.61-5.22) |
| Rajasthan | 4.17 (3.49-4.95) | 3.44 (2.82-4.15) | 3.42 (2.81-4.13) | 3.51 (2.89-4.22) | 4.32 (3.63-5.11) | 4.73 (4.01-5.55) |
| Sikkim | 54.10 (29.58-90.78) | 31.33 (13.52-61.74) | 47.69 (24.64-83.31) | 20.18 (6.55-47.11) | 32.81 (14.16-64.64) | 33.27 (14.36-65.57) |
| Tamil Nadu | 17.21 (15.61-18.93) | 19.39 (17.68-21.22) | 17.82 (16.18-19.58) | 16.72 (15.13-18.43) | 15.91 (14.35-17.58) | 17.10 (15.48-18.85) |
| Telangana | 12.06 (10.32-14.02) | 12.88 (11.07-14.91) | 10.06 (8.46-11.88) | 16.39 (14.31-18.69) | 16.51 (14.41-18.84) | 15.27 (13.23-17.53) |
| Tripura | 33.33 (24.58-44.20) | 23.00 (15.83-32.30) | 18.87 (12.43-27.45) | 39.21 (29.62-50.92) | 16.82 (10.78-25.03) | 23.83 (16.50-33.31) |
| Union Territories* (except Delhi) | 30.04 (20.92-41.78) | 25.64 (17.29-36.60) | 26.39 (17.93-37.46) | 22.90 (15.09-33.32) | 27.05 (18.50-38.18) | 23.60 (15.68-34.11) |
| Uttar Pradesh | 1.52 (1.29-1.78) | 1.67 (1.42-1.94) | 1.11 (0.92-1.34) | 1.54 (1.30-1.80) | 1.98 (1.71-2.27) | 1.94 (1.68-2.23) |
| Uttarakhand | 1.32 (0.48-2.87) | 1.53 (0.61-3.16) | 0.87 (0.23-2.24) | 1.31 (0.48-2.85) | 4.59 (2.84-7.02) | 5.25 (3.36-7.81) |
| West Bengal | 21.83 (20.32-23.42) | 15.11 (13.86-16.45) | 12.91 (11.75-14.15) | 15.17 (13.91-16.52) | 15.41 (14.13-16.77) | 14.85 (13.59-16.20) |
| ***Less Developed States*** | ***5.66 (5.38-5.95)*** | ***5.42 (5.15-5.70)*** | ***5.22 (4.96-5.50)*** | ***5.51 (5.24-5.78)*** | ***5.91 (5.63-6.20)*** | ***6.44 (6.15-6.73)*** |
| ***More Developed States*** | ***13.09 (12.62-13.57)*** | ***12.11 (11.66-12.57)*** | ***11.68 (11.24-12.14)*** | ***12.21 (11.76-12.68)*** | ***12.10 (11.64-12.56)*** | ***12.29 (11.83-12.77)*** |
| **India** | **9.04 (8.78-9.31)** | **8.42 (8.17-8.68)** | **8.10 (7.85-8.35)** | **8.45 (8.20-8.71)** | **8.62 (8.37-8.88)** | **8.98 (8.72-9.24)** |

*Union Territories (except Delhi) are not included in less or more developed states categories

## Table 4: Suicide rate ratios for 2017-2019 versus 2014-2016 (with 95% uncertainty interval (UI)) by sex and state

| **State** | **Total (95%UI)** | **Male (95% UI)** | **Female (95% UI)** |
| --- | --- | --- | --- |
| Andhra Pradesh | 0.88 (0.83-0.94) | 0.90 (0.81-0.98) | 0.87 (0.80-0.96) |
| Arunachal Pradesh | 0.41 (0.26-0.67) | 0.73 (0.35-1.53) | 0.26 (0.13-0.52) |
| Assam | 0.57 (0.52-0.64) | 0.58 (0.52-0.67) | 0.55 (0.47-0.65) |
| Bihar | 1.42 (1.19-1.69) | 1.44 (1.13-1.83) | 1.37 (1.07-1.74) |
| Chhattisgarh | 1.16 (1.08-1.24) | 1.19 (1.08-1.31) | 1.12 (1.01-1.24) |
| Delhi | 1.19 (1.06-1.34) | 1.14 (0.96-1.34) | 1.23 (1.05-1.45) |
| Goa | 1.20 (0.75-1.95) | 1.11 (0.50-2.26) | 1.25 (0.65-2.32) |
| Gujarat | 0.96 (0.89-1.03) | 0.86 (0.77-0.97) | 1.03 (0.94-1.13) |
| Haryana | 1.04 (0.94-1.18) | 1.01 (0.87-1.14) | 1.11 (0.92-1.35) |
| Himachal Pradesh | 1.60 (1.23-2.09) | 1.68 (1.09-2.45) | 1.55 (1.06-2.23) |
| Jammu & Kashmir and Ladakh | 0.66 (0.43-0.99) | 0.98 (0.52-1.96) | 0.44 (0.25-0.84) |
| Jharkhand | 2.06 (1.80-2.31) | 2.30 (1.97-2.70) | 1.73 (1.42-2.10) |
| Karnataka | 0.82 (0.76-0.88) | 0.73 (0.66-0.81) | 0.91 (0.83-1.01) |
| Kerala | 0.96 (0.86-1.06) | 1.01 (0.86-1.18) | 0.91 (0.79-1.04) |
| Madhya Pradesh | 1.11 (1.05-1.17) | 1.03 (0.95-1.11) | 1.16 (1.09-1.26) |
| Maharashtra | 1.07 (1.01-1.13) | 1.16 (1.06-1.27) | 1.00 (0.93-1.09) |
| Manipur | 1.01 (0.48-1.92) | 1.05 (0.24-4.64) | 0.86 (0.38-2.06) |
| Meghalaya | 1.06 (0.68-1.58) | 1.26 (0.72-2.22) | 0.80 (0.42-1.55) |
| Mizoram | 0.72 (0.37-1.34) | 0.89 (0.34-2.09) | 0.53 (0.21-1.44) |
| Nagaland | 1.12 (0.37-3.53) | 1.16 (0.19-6.15) | 0.70 (0.12-4.19) |
| Odisha | 0.87 (0.80-0.95) | 0.73 (0.64-0.83) | 1.02 (0.90-1.15) |
| Punjab | 1.22 (1.01-1.53) | 0.91 (0.69-1.20) | 1.84 (1.30-2.56) |
| Rajasthan | 1.12 (1.02-1.23) | 1.10 (0.99-1.24) | 1.14 (0.98-1.31) |
| Sikkim | 0.71 (0.46-1.06) | 0.80 (0.43-1.47) | 0.63 (0.35-1.07) |
| Tamil Nadu | 0.82 (0.78-0.87) | 0.72 (0.66-0.79) | 0.91 (0.84-0.99) |
| Telangana | 1.19 (1.09-1.31) | 1.00 (0.88-1.14) | 1.37 (1.22-1.54) |
| Tripura | 0.97 (0.79-1.17) | 0.87 (0.64-1.15) | 1.05 (0.81-1.34) |
| Union territories* (except Delhi) | 1.16 (0.94-1.45) | 1.65 (1.18-2.32) | 0.89 (0.65-1.21) |
| Uttar Pradesh | 1.19 (1.08-1.30) | 1.10 (0.97-1.26) | 1.25 (1.12-1.44) |
| Uttarakhand | 1.30 (0.90-1.87) | 0.59 (0.34-0.98) | 2.83 (1.63-5.11) |
| West Bengal | 0.85 (0.81-0.89) | 0.79 (0.73-0.85) | 0.91 (0.84-0.97) |
| ***Less Developed States*** | ***1.06 (1.03-1.09)*** | ***1.03 (0.99-1.07)*** | ***1.09 (1.05-1.14)*** |
| ***More Developed States*** | ***0.94 (0.92-0.96)*** | ***0.89 (0.86-0.92)*** | ***0.99 (0.96-1.02)*** |
| **India** | **0.98 (0.96-1.00)** | **0.94 (0.91-0.96)** | **1.01 (0.99-1.04)** |

*Union Territories (except Delhi) are not included in less or more developed states categories.
